# Long‐Term Outcomes of BK Viremia in Kidney Transplant Recipients

**DOI:** 10.1111/ctr.70333

**Published:** 2025-10-07

**Authors:** Srijan Tandukar, Qufei Wu, Sneha Chandrashekar, Divyash Shah, Chienhui Chiang, Kathryn Couch, Jennifer Trofe, Elizabeth M. Sonnenberg, Peter Abt, Mary Ann Lim, Vishnu Potluri, Roy Bloom

**Affiliations:** ^1^ Renal‐Electrolyte and Hypertension Division Hospital of the University of Pennsylvania Philadelphia USA; ^2^ Center for Clinical Epidemiology and Biostatistics University of Pennsylvania Philadelphia USA; ^3^ University of Pennsylvania Philadelphia USA; ^4^ Department of Surgery Hospital of the University of Pennsylvania Philadelphia USA; ^5^ Leonard Davis Institute of Health Economics University of Pennsylvania Philadelphia USA

**Keywords:** biopsy‐proven acute rejection, BK nephropathy, BK virus, de novo DSA, pre‐existing DSA

## Abstract

**Background:**

BK viremia is associated with worse kidney transplant function and de novo donor‐specific antibodies (DSA), but mortality and graft survival are not impacted in the short‐ and intermediate‐term. The long‐term impact of BK viremia remains unclear.

**Methods:**

We performed a single‐center retrospective study on 1058 consecutive kidney or kidney‐pancreas transplant recipients. We classified the cohort based on the presence or absence of BK viremia, BK viral loads, persistence of BK viremia, and pre‐existing or de novo DSA. Outcomes included mortality, graft loss, death‐censored graft survival (DCGS), estimated glomerular filtration rate, biopsy‐proven acute rejection (BPAR), de novo DSA, ureteral stenosis, and genitourinary (GU) malignancies.

**Results:**

Over a median follow‐up period of 9.7 years, there was no difference in graft loss (*p* = 0.08) and mortality (*p* = 0.56). Time‐varying multivariable analysis showed no difference in DCGS [HR 1.02, (0.91–1.16)]. Compared to never BK viremic patients, patients with BK viremia had similar graft function at 5 and 10 years (*p* = 0.09 and 0.65, respectively), rates of GU malignancies (7.0% vs. 5.2%, *p* = 0.35) and ureteral stenosis (0.8% vs. 0.4%, *p* = 0.63). Patients with BK viral loads >10 000 copies/mL had a higher risk of de novo DSA [HR 1.71, (1.08–2.68)] and BPAR [HR 2.11, (1.28–3.47)].

**Conclusions:**

BK viremia did not impact mortality, graft loss, kidney function, GU malignancies, and ureteral stenosis over long‐term follow‐up, but BPAR episodes and development of de novo DSA were higher with viral loads >10 000 copies/mL. Strict monitoring protocols and immunosuppression reduction strategies are effective in minimizing the risk associated with BK viremia.

**Abbreviations**
ABMR: Antibody‐mediated rejection
ANOVA: Analysis of variance
BKVAN: BK virus‐associated nephropathy
BPAR: Biopsy‐proven acute rejection
CI: Confidence interval
cPRA: Calculated panel reactive antibody
DCGS: Death‐censored graft survival
DSA: Donor‐specific antibody
eGFR: Estimated glomerular filtration rate
EHR: Electronic health record
GU: Genitourinary
HR: Hazard ratio
IQR: Interquartile range
IVIG: Intravenous immunoglobulin
MFI: Mean fluorescent intensity
PCR: Polymerase chain reaction
rATG: Rabbit anti‐thymocyte globulin
REDCap: Research electronic data capture
SPK: Simultaneous kidney‐pancreas transplant
SQL: Structured query language
TCMR: T cell‐mediated rejection

## 1 Introduction

BK virus is a double‐stranded DNA polyomavirus first identified in a kidney transplant recipient in 1971 [1, 2]. For immunocompetent individuals, BK virus typically remains latent in the uroepithelium, but reactivation can occur in immunocompromised patients [3, 4]. BK viremia has been reported in around 10%–30% of kidney transplant recipients with around 10% of viremic patients developing BK virus‐associated nephropathy (BKVAN) [5, 6, 7, 8, 9, 10].

Prior studies have shown increased risk of graft dysfunction with BKVAN and development of de novo donor‐specific antibody (DSA) in the short‐ and intermediate‐term, but long‐term outcomes are unknown [11, 12, 13]. BKVAN presents with tubulitis, interstitial inflammation, and eventual interstitial fibrosis on histology, often indistinguishable from an acute rejection except for the pathognomonic positive SV40 stain. Historically, as high as 60% of BK viremic patients developed severe BKVAN leading to graft failure, but with the advent of BK viral surveillance and protocolized immunosuppression modification in the modern era, the rates of BK viremia associated graft failures have declined. BK viremia has also been shown to be associated with a higher risk of genitourinary (GU) cancer and ureteral stenosis; however, long‐term prevalence is unknown [14, 15, 16, 17, 18].

This study aims to describe the association of BK viremia in kidney transplant recipients with long‐term outcomes of mortality, graft function and survival, biopsy‐proven acute rejection (BPAR), development of de novo DSA, and urologic complications.

## 2 Methods

### 2.1 Study Population

**FIGURE 1:**
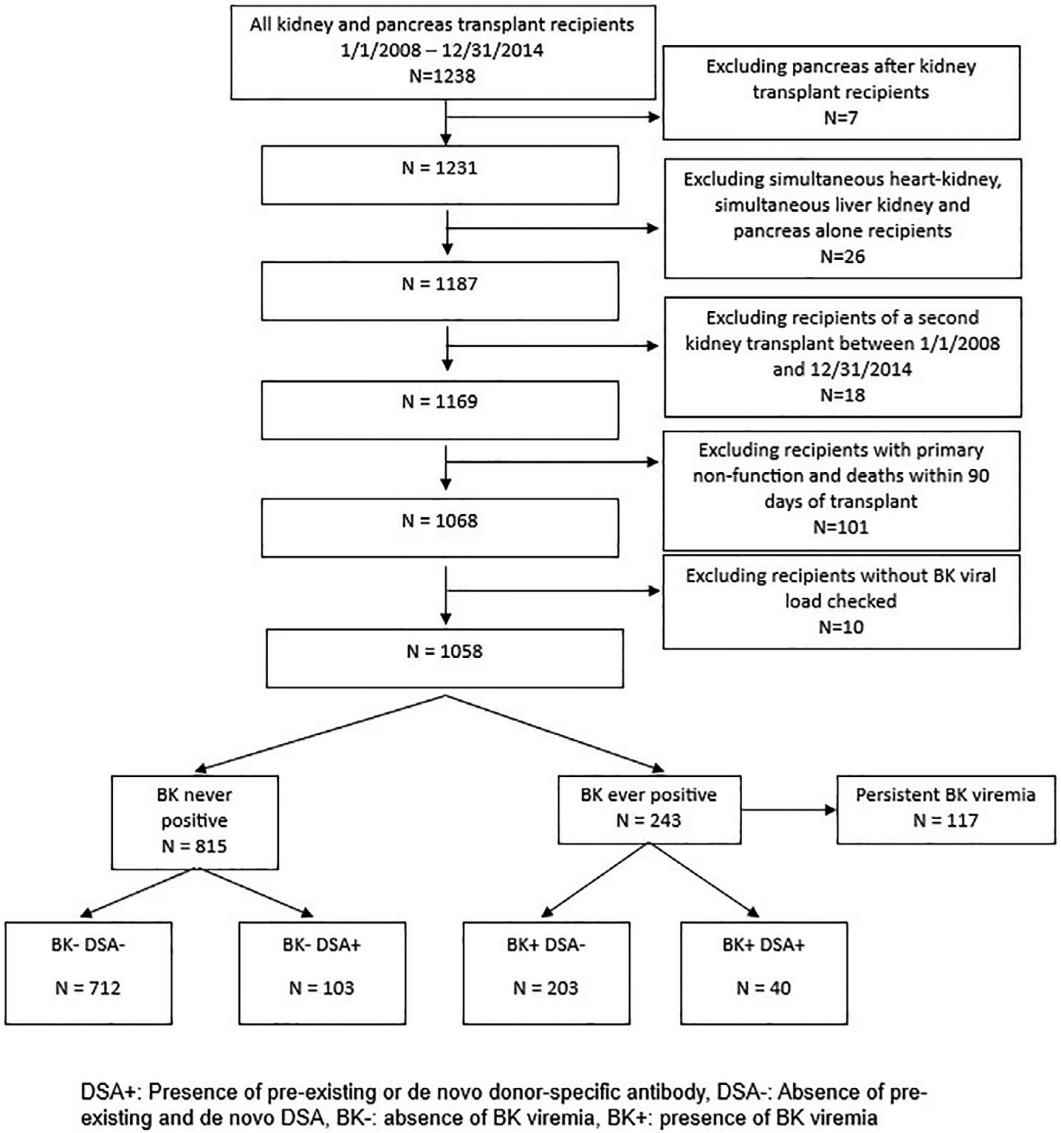
Flowchart showing cohort derivation for the study.

This is a retrospective study on kidney or simultaneous pancreas‐kidney (SPK) transplant recipients at the University of Pennsylvania between January 1, 2008, and December 31, 2014. The study was approved by the University of Pennsylvania Institutional Review Board (Protocol number 854001), and it adheres to the Declaration of Helsinki and Istanbul. Patients excluded from the study included multi‐organ transplant recipients other than SPK, recipients of a second kidney transplant in the same time interval between 2008 and 2014, patients with primary non‐function and death within 90 days of transplant, or those without any BK viral loads checked (Figure 1). Data were collected by directly querying the EPIC electronic health record (EHR) system using SQL coding and supplemented by manual chart reviews.

### 2.2 Immunosuppression Protocol

Induction immunosuppression was typically rabbit anti‐thymocyte globulin (rATG), with standard maintenance immunosuppression consisting of tacrolimus, mycophenolate mofetil, and prednisone (Table S1). The rATG (Thymoglobulin; Genzyme, Cambridge, MA) was dosed at 1.5 mg/kg of actual body weight intravenously over 4–6 h, first dose given intraoperatively before reperfusion and subsequent doses given inpatient or outpatient for a total of 3–5 doses based on immunologic risk. Basiliximab (Simulect, Novartis Pharmaceuticals Corp., East Hanover, NJ) was used instead (20 mg intraoperatively and on Day 3) for patients deemed to be of low immunologic risk. Patients with high calculated panel reactive antibodies (cPRA > 30%), prior transplant, and of African–American race were considered high risk and were typically administered five doses of rATG.

### 2.3 Diagnosis and Treatment of Rejection

For‐cause biopsies were performed in patients with an unexplained rise in serum creatinine (≥0.3 mg/dL above baseline) or proteinuria (>1 g/d). BPAR was diagnosed by a dedicated renal pathologist per the Banff 2005 classification. Banff borderline and 1a acute T cell‐mediated rejection (TCMR) was treated with solumedrol (three doses of 500 mg each). Banff grade 1b TCMR was treated with solumedrol with or without rATG (1–1.5 mg/kg, 1–3 doses) at the discretion of the treating nephrologist. Banff grade 2 or 3 acute TCMR was treated with rATG (1–1.5 mg/kg, 1–3 doses). Acute antibody‐mediated rejection (ABMR) was treated with plasmapheresis (3–4 sessions every other day) with 100 mg/kg of intravenous immunoglobulin (IVIG) (Gammagard, Baxter Healthcare Corp., Westlake Village, CA) after every plasmapheresis session and 500 mg/kg IVIG after the last plasmapheresis session. Rituximab (1–2 doses of 375 mg/m^2^, Rituxan; Genentech Inc., South San Francisco, CA) was used at the discretion of the treating nephrologist.

### 2.4 BK Virus Screening and Management

During the study period, the patients were screened for BK virus DNA by polymerase chain reaction (PCR) assay (Rotor‐Gene Q assay; Qiagen, Valencia, CA) at 3‐, 6‐, and 12‐months post‐transplant and then yearly thereafter. In September 2021, the detection assay was changed to the more sensitive Roche assay. The Roche method yielded comparable results, but was on average 0.3 log lower. Since this change was made around 7 years after the last transplant in the cohort, this small difference in test results is unlikely to have influenced the results of our analysis. Patients with detectable BK viremia had antimetabolite doses reduced by 50% with subsequent monitoring and discontinuation if levels further rose or persisted. If viral load increased despite antimetabolite discontinuation, the tacrolimus target level was progressively decreased until BK viral load improved. In some cases with persistent rise, tacrolimus was converted to cyclosporine at the discretion of the treating nephrologist. Serial BK viral loads were checked every 2–3 weeks after the detection of BK viremia with a level >4 log copies/mL (equivalent to 10 000 copies/mL), prompting a kidney transplant biopsy. BKVAN was diagnosed based on positive SV40 immunohistochemical staining. Antimetabolites were reintroduced after clearance of BK viremia at the discretion of the treating nephrologist.

### 2.5 Screening for DSAs

The presence of pre‐existing DSAs were noted from the HistoTrac software (Thermo Fisher Scientific) used by the HLA lab. During the study period, all kidney transplant recipients were screened for development of de novo DSA using single antigen Luminex assays (OneLambda, Canoga Park, CA) at 1‐, 3‐, 6‐, and 12‐months post‐transplant. Any DSA with mean fluorescent intensity (MFI) > 1000 was reported as positive. Detection of a positive DSA prompted dose increase of the antimetabolite or the calcineurin inhibitor at the discretion of the treating nephrologist.

### 2.6 Classification Based on BK Viremia and Presence of Pre‐Existing or De Novo DSA

The study cohort was divided into two categories based on presence of BK viremia as BK ever positive or BK never positive. Those patients who had persistence of BK viremia beyond 270 days post‐detection (50th percentile of BK viremia duration in the BK ever positive category) were categorized as persistent BK viremia. Patients with BK viremia were also stratified by BK viral load (<10 000 copies/mL vs. >10 000 copies/mL). The rationale for this was that most prior studies showed a higher incidence of BKVAN at a threshold of around 8000 to 10 000 copies/mL. Based on presence or absence of BK viremia and pre‐existing or de novo DSA, the cohort was divided into four categories: BK−/DSA−, BK−/DSA+, BK+/DSA−, and BK+/DSA+. Sensitivity analyses were conducted by excluding patients who had pre‐existing DSAs and patients who developed de novo DSA prior to the development of BK viremia.

### 2.7 Ureteral Stenosis and GU Malignancies

Data on ureteral stenosis and GU malignancies, including earliest date of diagnosis, were extracted through chart reviews by utilizing the EPIC search feature for specific terminologies. The timing of detection of GU malignancy in relation to transplant and the specific type of malignancy were also noted.

### 2.8 Statistical Analyses

Descriptive statistics were used to describe patient demographics. Continuous variables are presented as mean and standard deviation for parametric data and median and interquartile ranges for non‐parametric data. Categorical variables are presented as counts and proportions. Continuous variables were compared using *t*‐test or ANOVA for parametric data and Mann–Whitney *U* test or Kruskal–Wallis test for non‐parametric data. Survival analyses are presented as Kaplan–Meier curves. For multivariable regression analysis, Cox proportional hazards regression model was used to assess the outcomes of patient survival, graft survival, death‐censored graft survival (DCGS), de novo DSA development, and development of rejection post‐BK viremia. Variable selection was based on known variables that impact the outcomes being tested a priori and any variable found to have *p* value <0.1 in univariable analysis. The final model was selected after multiple iterations of backward selection and testing of best model fit following standard procedures. Data extraction from EPIC Clarity was done using Microsoft SQL Server. The data were ultimately imported into Research Electronic Data Capture (REDCap) with supplemental data entry via manual chart reviews. All statistical analyses were performed in R software, version 4.2.1. (R Foundation for Statistical Computing, Austria, Vienna).

## 3 Results

### 3.1 Baseline Characteristics

#### 3.1.1 Classification Based on Presence of BK Viremia

The recipients in the BK ever positive category were older (52.5 vs. 50.6 years, *p* = 0.05) and had a higher proportion of African American donors (21.4% vs. 12.6%, *p* < 0.001). Zero antigen mismatches were higher in the BK never positive group compared to BK ever positive (10.4% vs. 5.3%, *p* = 0.02) and persistent BK viremia groups (10.4% vs. 5.1%, *p* = 0.006). More patients in the BK ever positive group received rATG compared to the BK never positive group (89.3% vs. 82.7%, *p* = 0.02) (Table 1).

**TABLE 1:** Baseline characteristics of the cohort based on BK viremia.

Characteristics^c^	BK never positive (*N* = 815)	BK ever positive (*N* = 243)	*p* value^a^	Persistent BK viremia (*N* = 117)	*p* value^b^
**Recipient characteristics**					
Age	50.6 (13.3)	52.5 (12.1)	0.05	52.3 (12.4)	0.18
Race			0.15		0.3
Non‐African American	541 (66.4%)	149 (61.3%)		72 (61.5%)	
African American	274 (33.6%)	94 (38.7%)		45 (38.5%)	
Female gender	320 (39.3%)	93 (38.3%)	0.80	37 (31.6%)	0.11
Diabetes mellitus	282 (34.6%)	80 (32.9%)	0.63	34 (29.1%)	0.24
BMI	27.1 (5.1)	27.6 (5.3)	0.15	27.4 (5.4)	0.52
Dialysis at the time of transplant	643 (78.9%)	197 (81.1%)	0.46	47 (40.2%)	0.13
Native kidney disease			0.36		0.29
Diabetes mellitus	184 (22.6%)	52 (21.4%)		19 (16.2%)	
Hypertension	56 (6.9%)	21 (8.6%)		8 (6.8%)	
Polycystic kidney disease	72 (8.8%)	29 (11.9%)		15 (12.8%)	
Others	503 (61.7%)	141 (58%)		75 (64.1%)	
Blood group			0.02		0.08
A	321 (39.4%)	80 (32.9%)		39 (33.3%)	
AB	43 (5.3%)	12 (4.9%)		5 (4.3%)	
B	126 (15.5%)	28 (11.5%)		12 (10.3%)	
O	325 (39.9%)	123 (50.6%)		61 (52.1%)	
Rabbit antithymocyte globulin induction	674 (82.7%)	217 (89.3%)	0.02	105 (89.7%)	0.07
Peak PRA Class 1	46.2 (34.4)	42.0 (37.1)	0.42	50.5 (34.8)	0.55
Peak PRA Class 2	56.3 (31.5)	67.2 (30.7)	0.06	72.5 (25.6)	0.07
**Donor characteristics**					
Age	38.8 (15.7)	37.6 (15.5)	0.28	38.2 (15.2)	0.68
Race			<0.001		0.11
African American	103 (12.6%)	52 (21.4%)		21 (17.9%)	
Non‐African American	712 (87.4%)	191 (78.6%)		96 (82.1%)	
Female gender	397 (48.7%)	108 (44.4%)	0.29	48 (41.0%)	0.12
Diabetes mellitus	58 (7.1%)	22 (9.1%)	0.32	16 (13.7%)	0.01
BMI	26.7 (6.5)	26.8 (6.2)	0.80	27.7 (6.6)	0.1
Hypertension	148 (18.2%)	47 (19.3%)	0.35	25 (21.4%)	0.7
Terminal creatinine	1.1 (0.6)	1.1 (0.6)	0.6	1.1 (0.6)	0.38
HCV antibody positive	0 (0.0%)	1 (0.4%)	0.28	1 (0.9%)	0.04
Cause of donor death			0.85		0.78
Anoxia	194 (34.7%)	60 (33.5%)		26 (30.2%)	
Cerebrovascular/Stroke	171 (30.6%)	58 (32.4%)		29 (33.7%)	
Head trauma	184 (32.9%)	58 (32.4%)		29 (33.7%)	
CNS tumor	3 (0.5%)	0 (0.0%)		0 (0%)	
Other	7 (1.3%)	3 (1.7%)		2 (2.3%)	
Blood group			0.26		0.8
A	288 (35.3%)	67 (27.5%)		34 (29.1%)	
AB	22 (2.7%)	7 (2.8%)		3 (2.6%)	
B	100 (12.2%)	25 (10.3%)		12 (10.3%)	
O	405 (49.7%)	144 (59.3%)		68 (58.1%)	
Kidney donor profile index			0.6		0.87
0%–20%	148 (26.6%)	54 (30.2%)		26 (30.2%)	
21%–35%	94 (16.9%)	33 (18.4%)		14 (16.3%)	
35%–85%	279 (50.2%)	84 (46.9%)		42 (48.8%)	
86%–100%	35 (6.3%)	8 (4.5%)		4 (4.7%)	
Type of donor			0.23		0.22
Living donor	256 (31.4%)	64 (26.3%)		31 (26.5%)	
Donation after brain death	485 (59.5%)	151 (62.1%)		70 (59.8%)	
Donation after circulatory death	74 (9.1%)	28 (11.5%)		16 (13.7%)	
**Transplant characteristics**					
Cold ischemia time (h)	13.2 (6.8)	12.9 (6.7)	0.59	13.5 (6.3)	0.73
HLA mismatch			0.02		0.006
0	85 (10.4%)	13 (5.3%)		6 (5.1%)	
1	15 (1.8%)	1 (0.4%)		0 (0%)	
2	56 (6.9%)	14 (5.8%)		3 (2.8%)	
3	147 (18.0%)	35 (14.4%)		14 (12.0%)	
4	181 (22.2%)	72 (29.6%)		41 (35.0%)	
5	224 (27.5%)	78 (32.1%)		37 (31.6%)	
6	107 (13.1%)	30 (12.3%)		16 (13.7%)	
Organ transplanted			0.49		0.9
Kidney transplant alone	789 (96.8%)	233 (95.9%)		113 (96.6%)	
Simultaneous kidney‐pancreas transplant	26 (3.2%)	10 (4.1%)		4 (3.4%)	

^a^ *p* value for comparison of BK never positive and BK ever positive groups.

^b^ *p* value for comparison of BK never positive and Persistent BK viremia groups.

^c^ Continuous data and categorical data are presented as mean, SD, and count (%), respectively, unless stated otherwise.

#### 3.1.2 Classification Based on BK Viremia and Pre‐Existing or De Novo DSA

The BK−/DSA− category had the youngest recipients, whereas BK+/DSA+ had the oldest recipients (45 vs. 51.5 years, *p* < 0.001). The BK+/DSA+ category had the largest proportion of African American recipients (57.5%) and donors (22.5%) (*p* < 0.001 and <0.01, respectively). The BK−/DSA− category had the highest proportion of zero HLA mismatches (11.8%) whereas the BK+/DSA+ category had the highest proportion of six HLA mismatches (20%) (*p* < 0.01) (Table 2).

**TABLE 2:** Baseline characteristics of the cohort based on BK viremia and pre‐existing or de novo donor‐specific antibody.

Characteristics^a^	BK−/DSA− (*N* = 712)	BK−/DSA+ (*N* = 103)	BK+/DSA− (*N* = 203)	BK+/DSA+ (*N* = 40)	*p* value
**Recipient characteristics**					
Age	51.4 (13.1)	45 (13.3)	52.7 (12.3)	51.5 (11.4)	<0.001
Race					<0.001
Non‐African American	492 (69.1%)	49 (47.6%)	132 (65.0%)	17 (42.5%)	
African American	220 (30.9%)	54 (52.4%)	71 (35.0%)	23 (57.5%)	
Female gender	281 (39.5%)	39 (37.9%)	84 (41.4%)	9 (22.5%)	0.20
Diabetes mellitus	250 (35.1%)	32 (31.1%)	69 (34%)	11 (27.5%)	0.68
BMI (kg/m^2^)	27.0 (5.1)	27.3 (5.3)	27.5 (5.5)	28.0 (4.2)	0.44
Dialysis at the time of transplant	559 (78.5%)	84 (81.6%)	165 (81.3%)	31 (80%)	0.78
Native kidney disease					0.1
Diabetes mellitus	163 (22.9%)	21 (20.4%)	47 (23.2%)	5 (12.5%)	
Hypertension	44 (6.2%)	12 (11.7%)	16 (7.9%)	5 (12.5%)	
Polycystic kidney disease	69 (9.7%)	3 (2.9%)	24 (11.8%)	5 (12.5%)	
Others	436 (61.2%)	67 (65%)	116 (57.1%)	25 (62.5%)	
Blood group					0.13
A	290 (40.7%)	31 (30.1%)	67 (33.0%)	13 (32.5%)	
AB	37 (5.2%)	6 (5.8%)	10 (4.9%)	2 (5%)	
B	106 (14.9%)	20 (19.4%)	23 (11.3%)	5 (12.5%)	
O	279 (39.2%)	46 (44.7%)	103 (50.7%)	22 (50%)	
Rabbit antithymocyte globulin induction	588 (82.6%)	86 (83.5%)	181 (89.2%)	36 (90%)	0.1
Peak PRA Class I (%)	44.4 (34.1)	59.4 (33.9)	43.6 (37.5)	29.7 (33.4)	0.15
Peak PRA Class II (%)	57.8 (31.3)	48.5 (32.3)	65.2 (31.4)	77.3 (26.9)	0.12
**Donor characteristics**					
Age	39.1 (15.7)	36.6 (15.1)	37.6 (15.8)	37.5 (14.1)	0.33
Gender	353 (49.6%)	44 (42.7%)	87 (42.9%)	21 (52.5%)	0.19
Race					<0.01
Non‐African American	625 (87.8%)	87 (84.5%)	160 (78.8%)	31 (77.5%)	
African American	87 (12.2%)	16 (15.5%)	43 (21.2%)	9 (22.5%)	
Diabetes mellitus	55 (7.7%)	3 (2.9%)	19 (9.4%)	3 (7.5%)	0.25
BMI (kg/m^2^)	26.9 (6.7)	25.6 (4.8)	26.9 (6.3)	26.2 (5.5)	0.25
Hypertension	136 (19.1%)	12 (11.7%)	42 (20.7%)	5 (12.5%)	0.31
Terminal creatinine (mg/dL)	1.1 (0.6)	0.9 (0.5)	1.1 (0.6)	1.1 (0.7)	0.25
HCV antibody positive	0 (0%)	0 (0.0%)	1 (0.5%)	0 (0%)	0.41
Cause of death in donor					0.98
Anoxia	170 (34.8%)	24 (34.3%)	50 (33.6%)	10 (33.3%)	
Cerebrovascular/Stroke	151 (30.9%)	20 (28.6%)	48 (32.2%)	10 (33.3%)	
Head trauma	158 (32.3%)	26 (37.1%)	48 (32.2%)	10 (33.3%)	
CNS tumor	3 (0.6%)	0 (0.0%)	0 (0.0%)	0 (0%)	
Others	7 (1.4%)	0 (0.0%)	3 (2.0%)	0 (0%)	
Donor blood group					0.5
A	258 (36.1%)	30 (29.1%)	58 (28.6%)	9 (22.5%)	
AB	17 (2.4%)	5 (4.8%)	6 (3%)	1 (2.5%)	
B	82 (11.5%)	18 (17.5%)	20 (9.9%)	5 (12.5%)	
O	355 (49.9%)	50 (48.5%)	119 (58.6%)	25 (62.5%)	
Kidney donor profile index					0.31
0%–20%	122 (25.1%)	26 (37.1%)	42 (28.2%)	12 (40.0%)	
21%–35%	85 (17.5%)	9 (12.9%)	27 (18.1%)	6 (20.0%)	
35%–85%	246 (50.6%)	33 (47.1%)	74 (49.7%)	10 (33.3%)	
86%–100%	33 (6.8%)	2 (2.9%)	6 (4.0%)	2 (6.7%)	
Type of donor					0.67
Living donor	223 (31.3%)	33 (32.0%)	54 (26.6%)	10 (25.0%)	
Donation after brain death	424 (59.6%	61 (59.2%)	124 (61.1%)	27 (67.5%)	
Donation after circulatory death	65 (9.1%)	9 (8.7%)	25 (12.3%)	3 (7.5%)	
**Transplant characteristics**					
Cold ischemia time (h)	13.3 (6.9)	12.7 (6.3)	13.0 (6.8)	12.5 (6.2)	0.81
Number of HLA mismatches					<0.01
0	84 (11.8%)	1 (1.0%)	11 (5.4%)	2 (5%)	
1	14 (2.0%)	1 (1.0%)	1 (0.5%)	0 (0%)	
2	50 (7.0%)	6 (5.8%)	11 (5.4%)	3 (7.5%)	
3	130 (18.3%)	17 (16.5%)	32 (15.8%)	3 (7.5%)	
4	155 (21.8%)	26 (25.2%)	64 (31.5%)	8 (20%)	
5	188 (26.4%)	36 (35.0%)	62 (30.5%)	16 (40%)	
6	91 (12.8%)	16 (15.5%)	22 (10.8%)	8 (20%)	
Organ transplanted					0.06
Kidney transplant alone	693 (97.3%)	96 (93.2%)	193 (95.1%)	40 (100.0%)	
Simultaneous kidney‐pancreas transplant	19 (2.7%)	7 (6.8%)	10 (4.9%)	0 (0.0%)	

^a^ Continuous data and categorical data are presented as mean (SD) and count (%), respectively, unless stated otherwise.

#### 3.1.3 Classification Based on BK Viral Load

The cohort was divided into three groups based on BK viral load as BK never positive (*N* = 815), BK viral load <10 000 copies/mL (*N* = 117), and BK viral load >10 000 copies/mL (*N* = 126) groups (Table S2).

### 3.2 Kidney Graft Survival

**FIGURE 2:**
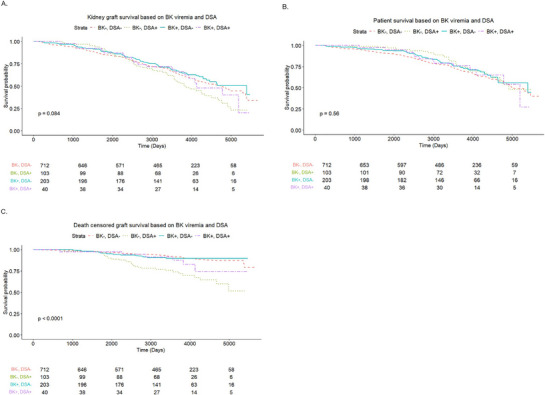
Kaplan–Meier curves showing (A) kidney graft survival, (B) patient survival, and (C) death‐censored graft survival based on BK viremia and DSA.

There was no difference in kidney allograft survival among the categories stratified by presence or absence of BK viremia and DSA on Kaplan–Meier analysis (*p* = 0.08) (Figure 2A) and on multivariable analysis [HR for BK−/DSA+ 1.34 (1–1.79), *p* = 0.05; BK+/DSA− 0.81 (0.63–1.04), *p* = 0.1; BK+/DSA+ 1 (0.63–1.59), *p* = 0.99; reference category: BK−/DSA−]. Older recipient age (age > 60) [HR 2.06 (1.69–2.51), *p* < 0.001], a history of pre‐transplant dialysis [HR 1.74 (1.31–2.30), *p* < 0.001], diabetes in recipient [HR 1.61 (1.33–1.94), *p* < 0.001] and BPAR [HR 2.04 (1.56–2.67), *p* < 0.001] was associated with higher risk of graft loss on multivariable analysis (Table 3A). There was no difference in graft survival among patients with greater or less than 10 000 copies/mL of BK viremia and BK never positive groups (*p* = 0.47) (Figure S1A).

**TABLE 3:** Multivariable Cox regression analysis.

A. Overall graft loss				
Variable	Categories	*N*	Hazard ratio	*p* value
BK and DSA category	BK‐ / DSA‐	712	Reference	
	BK‐/DSA+	103	1.34 (1, 1.79)	0.05
	BK+/DSA‐	203	0.81 (0.63, 1.04)	0.1
	BK+/DSA+	40	1 (0.63, 1.59)	0.99
Recipient age	Age < 60 years	782	Reference	
	Age >60 years	276	2.06 (1.69, 2.51)	<0.001
Recipient ethnicity	Non African American	690	Reference	
	African American	368	1.2 (0.98, 1.46)	0.07
Recipient diabetes mellitus	No DM/ Unknown status	696	Reference	
	DM	362	1.61 (1.33, 1.94)	<0.001
Dialysis status at transplant	Not on dialysis	218	Reference	
	On dialysis	840	1.74 (1.31, 2.3)	<0.001
HLA mismatch	>0 HLA mismatch	960	Reference	
	0 HLA mismatch	98	0.97 (0.69, 1.36)	0.85
Acute rejection	No acute rejection	954	Reference	
	Acute rejection	104	2.04 (1.56, 2.67)	<0.001

B. Mortality				
Variable	Categories	*N*	Hazard ratio	*p* value
BK and DSA category	BK‐ / DSA‐	712	Reference	
	BK‐ / DSA+	103	0.87 (0.58, 1.29)	0.48
	BK+/DSA‐	203	0.80 (0.60, 1.07)	0.13
	BK+/DSA+	40	0.96 (0.55, 1.69)	0.89
Recipient age	Age < 60 years	782	Reference	
	Age >60 years	276	2.86 (2.30, 3.57)	<0.001
Recipient ethnicity	Non African American	690	Reference	
	African American	368	0.98 (0.78, 1.24)	0.87
Recipient diabetes mellitus	No DM/ Unknown status	696	Reference	
	DM	362	1.94 (1.56, 2.40)	<0.001
Dialysis status at transplant	Not on dialysis	218	Reference	
	On dialysis	840	1.72 (1.25, 2.36)	<0.001
HLA mismatch	>0 HLA mismatch	960	Reference	
	0 HLA mismatch	98	0.82 (0.55, 1.23)	0.34
Acute rejection	No acute rejection	954	Reference	
	Acute rejection	104	1.41 (0.99, 2.02)	0.06

C. Time varying analysis for death‐censored graft loss						
	Univariate			Multivariate		
Variable	HR	*p* value	95% CI	HR	*p* value	95% CI
Time varying BK viremia status	0.97	0.6	0.98 – 1.01	1.02	0.68	0.9 – 1.16
Age >60 years	0.35	<0.001	0.28 – 0.39	0.4	<0.001	0.34 ‐ 0.47
Recipient African American race	2.1	<0.001	1.85 – 2.26	2.11	<0.001	1.89 – 2.36
On dialysis pre‐transplant	1.29	<0.001	1.15 ‐ 1.41	1.59	<0.001	1.36 – 1.85
Recipient diabetes mellitus	0.78	<0.001	0.69 – 0.86	0.57	<0.001	0.51 – 0.65
Zero HLA mismatch	1.42	<0.001	1.22 – 1.66	1.87	<0.001	1.59 – 2.21
Biopsy‐proven acute rejection	3.32	<0.001	2.97 – 3.72	2.76	<0.001	2.46 – 3.12

D. Development of de novo DSA				
Variable	Categories	*N*	Hazard ratio	*p* value
BK category	BK never positive	807	Reference	
	BK <10000 copies/ml	115	0.98 (0.56, 1.72)	0.94
	BK >10000 copies/ml	126	1.71 (1.08, 2.68)	0.02
Recipient age	Age < 60 years	773	Reference	
	Age >60 years	275	0.54 (0.34, 0.86)	0.01
Recipient ethnicity	Non African American	689	Reference	
	African American	359	1.69 (1.17, 2.44)	0.006
Recipient diabetes mellitus	No DM/ Unknown status	688	Reference	
	DM	360	0.85 (0.58, 1.24)	0.39
Dialysis status at transplant	Not on dialysis	216	Reference	
	On dialysis	832	0.89 (0.56, 1.40)	0.6
HLA mismatch	>0 HLA mismatch	950	Reference	
	0 HLA mismatch	98	0.25 (0.08, 0.79)	0.02
Acute rejection	No acute rejection	945	Reference	
	Acute rejection	103	1.88 (1.21, 2.93)	0.005

E. Biopsy‐proven acute rejection episodes				
Variable	Categories	*N*	Hazard ratio	*p* value
BK category	BK never positive	815	Reference	
	BK <10000 copies/ml	117	1.19 (0.64, 2.21)	0.57
	BK >10000 copies/ml	126	2.11 (1.28, 3.47)	0.003
Recipient age	Age < 60 years	782	Reference	
	Age >60 years	276	0.62 (0.36, 1.05)	0.07
Recipient ethnicity	Non African American	690	Reference	
	African American	368	2 (1.32, 3.05)	0.001
Recipient diabetes mellitus	No DM/ Unknown status	696	Reference	
	DM	362	1.37 (0.91, 2.06)	0.13
Dialysis status at transplant	Not on dialysis	218	Reference	
	On dialysis	840	1.12 (0.64, 1.94)	0.7
HLA mismatch	>0 HLA mismatch	960	Reference	
	0 HLA mismatch	98	1.16 (0.55, 2.46)	0.69
Pre‐formed or de novo DSA	No acute rejection	915	Reference	
	Acute rejection	143	1.6 (1, 2.55)	0.048

### 3.3 Patient Survival

The patient survival was not different between the categories stratified by presence or absence of BK viremia and DSA on Kaplan–Meier analysis (*p* = 0.56) (Figure 2B) and on multivariable analysis [HR for BK−/DSA+ 0.87 (0.58–1.29), *p* = 0.48; BK+/DSA− 0.8 (0.60–1.07), *p* = 0.13; BK+/DSA+ 0.96 (0.55–1.69), *p* = 0.89; reference category: BK−/DSA−]. There was a higher risk of mortality in patients with recipient age >60 years [HR 2.86 (2.30–3.57), *p* < 0.001], diabetes in recipient [HR 1.94 (1.56–2.40), *p* < 0.001] and a history of pre‐transplant dialysis [HR 1.72 (1.25–2.36), *p* < 0.001] (Table 3B). There was no difference in patient survival among patients with greater or less than 10 000 copies/mL of BK viremia and BK never positive groups (*p* = 0.69) (Figure S1B).

### 3.4 DCGS

The DCGS was worst for the BK−/DSA+ group on Kaplan–Meier analysis (*p* < 0.0001) (Figure 2C). This was also true on multivariable analysis with BK−/DSA+ group having a higher risk of death‐censored graft loss [HR 2.53 (1.58–4.03), *p* < 0.001] compared to the other groups. Patients of African–American race [HR 2.04 (1.35–3.08), *p* < 0.001] and those with BPAR episode [HR 3.04 (1.97–4.70), *p* < 0.001] had higher risk of death‐censored graft loss (Table S3). Presence of BK viremia also did not show a significant difference in a time varying univariable (HR 0.97, 95% CI 0.98–1.01) and multivariable (HR 1.02, 95% CI 0.9–1.16) analysis (Table 3C). There was no difference in DCGS in patients with BK viremia <10 000 copies/mL versus >10 000 copies/mL as compared to BK never positive group (*p* = 0.54) (Figure S1C).

### 3.5 Kidney Graft Function

**FIGURE 3:**
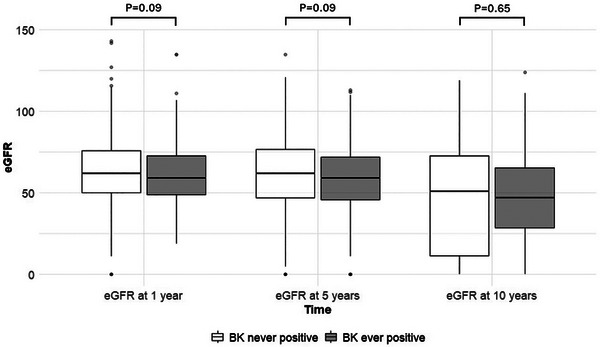
Comparison of eGFR values at different time points post‐transplant.

There was no difference in estimated glomerular filtration rate (eGFR) when compared between BK ever positive and never positive groups at 1‐year (*p* = 0.09), 5‐year (*p* = 0.09), and 10‐year (*p* = 0.65) time points (Figure 3).

### 3.6 BK Viremia and BK Nephropathy

The median BK viral load was 1950 copies/mL (IQR 650–7630) with a maximum reported viral load of 39 000 000 copies/mL. The duration of positive BK viral load was a median of 270 days (IQR 63–770) from the earliest to the last detectable level. The earliest detection of BK viremia was at a median posttransplant day of 240 (IQR 116–665). BKVAN was diagnosed in 3.3% (*N* = 8) of BK viremic patients based on a positive SV40 stain by immunohistochemistry (Tables 3B and 4, Figure S2).

**TABLE 4:** Long‐term outcomes of BK viremia.

Outcome	BK ever positive *N* = 243	BK never positive *N* = 815	*p* value
Genitourinary malignancies	17 (7%)	42 (5.2%)	0.35
Prostate	4 (1.7%)	8 (0.9%)	
Kidney	9 (3.7%)	24 (2.9%)	
Prostate and kidney	1 (0.4%)	3 (0.3%)	
Urinary bladder	1 (0.4%)	5 (0.6%)	
Ureter	1 (0.4%)	0 (0%)	
Other urothelial cancers	1 (0.4%)	2 (0.2%)	
Ureteral stenosis	2 (0.8%)	4 (0.4%)	0.63
Diagnosed <1 year post‐transplant	1 (0.4%)	1 (0.1%)	
Diagnosed >1 year post‐transplant	1 (0.4%)	3 (0.3%)	
BK nephropathy (with positive BK stain)^a^	8 (3.3%)	−	−
BK viral load, copies/mL	1950 (650–7630)	−	−
Duration of positive BK viremia, days	270 (63–770)	−	−
Earliest detection of BK viremia, days posttransplant	240 (116–665)	−	−
Biopsy‐proven acute rejection	33 (13.6%)	74 (9.1%)	0.05
Borderline rejection	12 (4.9%)	30 (3.7%)	0.72
Banff 1a rejection	6 (2.5%)	15 (1.8%)	0.93
Banff 1b rejection	7 (2.9%)	12 (1.5%)	0.8
Banff 2a rejection	4 (1.6%)	7 (0.9 %)	0.74
Banff 2b rejection	0 (0%)	1 (0.1%)	1
Banff 3 rejection	1 (0.4 %)	1 (0.1%)	0.54
Antibody‐mediated rejection	3 (1.2%)	8 (0.9%)	1

^a^ The viral loads on patients who had BKVAN were as follows at the time of their biopsy: 706 540, 675 000, 385 000, 245 774, 70 850, 38 025, 23 725, 23 100 copies/mL.

### 3.7 Pre‐Existing and De Novo DSA

Of 143 patients with a positive DSA test, 133 patients had de novo DSA, whereas 10 patients had pre‐existing DSAs. All patients with pre‐existing DSAs also had positive DSAs during surveillance checks post‐transplant at least on one occasion. Class I DSA was detected in 6.2% and 5.8% (*p* = 0.94), and Class II DSA in 13.6% and 10.6% (*p* = 0.23) among BK ever positive and BK never positive groups, respectively (Figures S3 and S4, Tables S4 and S5). On multivariable regression analysis, the development of de novo DSA did not differ between BK ever positive and BK never positive groups (*p* = 0.13) (Table S6)

### 3.8 Timing of BK Viremia in Relation to De Novo DSA Detection

There were 40 patients who developed both BK viremia and de novo DSA. Among them, 37 patients developed BK viremia before de novo DSA detection, two developed it after detection, and one developed it concurrently. For most cases, BK viremia tended to precede the development of de novo DSA, although both were routinely checked post‐transplant.

### 3.9 BPAR Episodes

**FIGURE 4:**
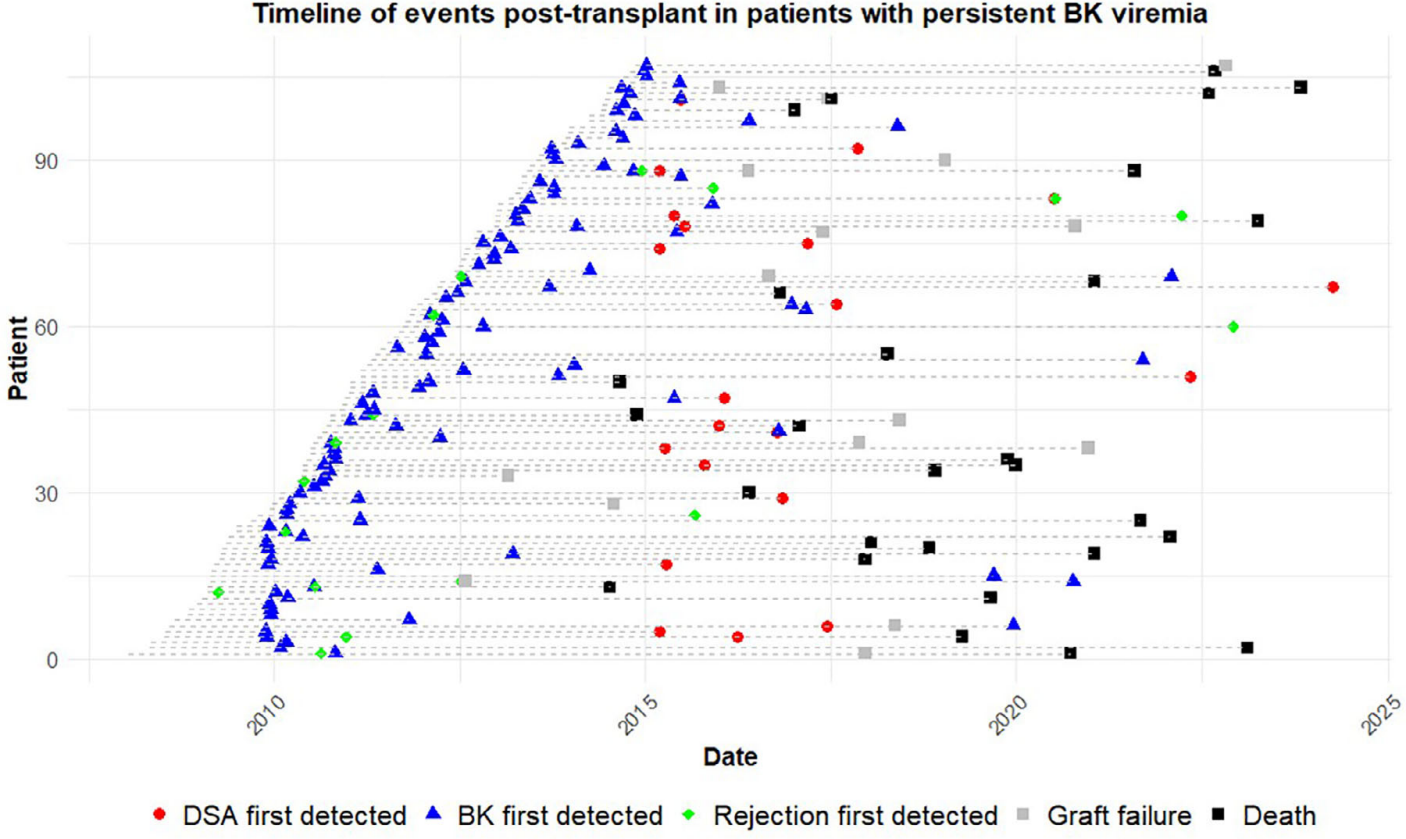
Timeline of events post‐transplant in recipients with persistent BK viremia.

Kidney transplant biopsies were performed for cause in 65 patients (26.7%) with BK viremia. BPAR was detected in 33 patients (13.6%) in BK ever positive group and 74 patients (9.1%) in BK never positive group. BPAR was less common in the BK never positive group [HR 0.61 (0.4–0.92), *p* = 0.02] compared to BK ever positive group on multivariable regression analysis (Table S7). The timing of the first episode of BPAR in relation to the development of BK viremia, first detection of de novo DSA, graft failure, and death are shown for the 117 patients with persistent BK viremia group in Figure 4.

### 3.10 Timing of BK Viremia and Acute Rejection Episodes

There were 33 patients (14.6%) among the 243 patients who were in the BK ever positive category who developed BPAR (Tables 4 and S8). Eight patients (3.3%) with BK viremia developed BK virus nephropathy (defined as a positive SV40 stain on biopsy). Among these eight, only three patients (1.2%) had concurrent rejection and BK virus nephropathy.

### 3.11 De Novo DSA and Kidney Biopsies

Of the 133 patients who developed de novo DSA, 51 underwent a kidney biopsy, among whom 26 had biopsy‐proven rejection but none of the patients had a positive SV40 staining (Table S8). Among those with rejection, 16 patients had T cell‐mediated rejection, two had antibody‐mediated rejection, and eight had mixed rejection. Treatments for these patients are summarized in Table S9.

### 3.12 Association of BK Viral Load With De Novo DSA and Acute Rejection

On subgroup analysis, BK viral load >10 000 copies/mL was associated with a higher risk of de novo DSA development [HR 1.71 (1.08–2.68)] (Table 3D) and a higher risk of BPAR episodes [HR 2.11 (1.28–3.47)] (Table 3E).

### 3.13 Treatment of BK Viremia

Among those with BK viremia, mycophenolate mofetil or mycophenolic acid was reduced by 50% in 25 patients, held in 135; azathioprine dose was reduced in seven patients and held in two. Those patients who had the anti‐metabolite held did not have a higher risk of de novo DSA development (*p* = 0.3) (Table S10).

Chart review showed that 136 patients (12.6%) received at least one additional therapy (Intravenous immunoglobulin or IVIG, leflunomide, cidofovir, levofloxacin, or ciprofloxacin) after the detection of BK viremia. Only five patients received the medications exclusively for BK viremia (IVIG in one patient, levofloxacin for one patient, and ciprofloxacin for three patients). For the remaining 131, IVIG was used for rejection in one patient, and the rest received either levofloxacin or ciprofloxacin for some type of concurrent infection (but not specifically BK viremia), predominantly UTI or pyelonephritis, but also infections at other sites. None of the patients received leflunomide or cidofovir.

### 3.14 Sensitivity Analyses

Sensitivity analyses that excluded pre‐existing DSAs from the cohort (*N* = 10) and those who developed de novo DSA before the onset of BK viremia (*N* = 2) or concurrently with BK viremia (*N* = 1) led to the same overall conclusions as presented above.

### 3.15 Ureteral Stenosis

There was no difference in the occurrence of ureteral stenosis between BK ever positive and never positive groups (0.8% vs. 0.4%, *p* = 0.63). Only one patient in each group developed it within the first year of transplant, and the overall incidence was low (Table 4).

### 3.16 GU Malignancies

GU malignancies developed in 17 (7%) patients in BK ever positive group and in 42 (5.2%) in BK never positive group (*p* = 0.35). The highest incidence was of renal cell carcinoma occurring in 3.7% and 2.9%, respectively (Table 4).

## 4 Discussion

There was no evidence of increased mortality and graft loss in patients with BK viremia with or without presence of DSA compared to those without BK viremia over a median of 9.7 years of follow‐up. Overall incidence of BK viremia in our cohort of kidney and kidney‐pancreas transplant patients was 22.9% with biopsy‐confirmed BK virus nephropathy in 3.3%. These findings were consistent for DCGS using time‐varying analysis and for those with higher grades of BK viremia, defined as above 10 000 copies/mL in our study. With protocolized screening for BK viremia and reduction of immunosuppression with BK viremia detection, eGFR at 5 and 10 years posttransplant was similar between those with and without BK viremia. There was a higher risk of BPAR in patients with BK viremia, but there was no difference in the development of de novo DSA in patients with and without BK viremia. In subgroup analyses, we found that BK viral load >10 000 copies/mL was associated with a higher risk of BPAR and de novo DSA development. The incidence of GU malignancies and ureteral stenosis was similar between the groups. This study includes a large cohort of patients with the longest follow‐up to date reported in the published literature with standardized screening and immunosuppression reduction protocols and inclusion of granular GU malignancy and ureteral stenosis data.

Detection of BK viremia has been associated with worse graft function in some studies with short‐ and intermediate‐term follow‐up [4, 9, 11, 13]. Over a follow‐up duration of 3 years, Sawinski et al. previously reported no increase in the risk of graft loss [12]. Similarly, Hardinger et al. reported similar DCGS in patients with BK viremia compared to those without over a 5‐year follow‐up [19]. Our study demonstrated no increase in patient and graft loss over a follow‐up period of 9.7 years. To our knowledge, this is the longest duration of follow‐up ever reported.

Latest BK virus screening guidelines published in 2024 recommend screening of BK viremia using PCR testing monthly until month 9, then every 3 months until 2 years posttransplant (strong recommendation, high level evidence). The consensus committee comprising of international experts from multiple institutions also recommended checking BK viral loads within 2–3 weeks when the viral loads are detected at a level of 1000–10 000 copies/mL [20]. Our screening protocol during the study from 2008 to 2014 was less frequent than what is currently recommended with BK viral load checks at 3, 6, and 12 months. A positive BK viral load prompted serial checks every 2–3 weeks. Our study suggests that a less frequent screening protocol for BK viremia may be feasible without compromising graft and patient outcomes.

The cornerstone of BK viremia management is early detection and lowering of immunosuppressive regimen as recommended by latest consensus committee guidelines (strong recommendation, medium evidence) [20]. The same approach was taken for BK viremia management in our cohort with reduction of antimetabolite as the first step, followed by discontinuation if levels progressively rise. In the absence of effective control of BK viremia despite these measures, lowering of tacrolimus trough and a switch to cyclosporine were subsequent steps taken to ameliorate BK viremia in a stepwise manner. Moreover, we did not find an increased risk of de novo DSA development after stopping antimetabolites in our study. Our approach to management is supported by multiple studies showing the effectiveness of immunosuppression reduction in controlling BK viremia and prevention of BK virus nephropathy [12, 19]. Although a minority of patients received other therapies (IVIG, levofloxacin or ciprofloxacin), consistent improvements were found across the cohort in BK viremia with immunosuppression reduction alone. Early recognition of BK viremia and immunosuppression reduction strategies may have helped preserve kidney allograft function in BK viremic patients in the new era compared to previous eras with a relatively low incidence of BKVAN in our cohort.

The findings in our study are concordant with those in earlier studies that showed a strong association of BPAR and BK viremia [21, 22, 23, 24, 25]. The pathological findings of BKVAN may mimic those of BPAR with tubulointerstitial inflammation of different degrees. The diagnosis of BKVAN is made based on a positive SV40 stain with diagnostic accuracy further strengthened by the finding of viral cytopathic changes in the tubular cells [26]. We did not see a difference in development of de novo DSA between those with and without BK viremia after adjusting for BPAR and HLA mismatches among other covariates. However, patients with BK viral loads >10 000 copies/mL were found to have a higher risk of developing de novo DSA and BPAR episodes. Our findings are similar to findings from some prior studies that showed a higher prevalence of de novo DSA in some subgroups of BK viremic patients [12, 27].

BK virus infection has been shown to be associated with late‐onset ureteral stenosis, defined as ureteral stenosis occurring >1‐month posttransplant [14, 15, 28, 29]. This may present clinically as a decrease in eGFR with imaging evidence of hydroureteronephrosis of varying degrees. In our cohort, there was an overall low incidence of ureteral stenosis with occurrence in only six patients (0.6%). Prior studies have shown an increased incidence of GU malignancies among kidney transplant recipients with BK virus infection [16, 17]. These findings are supported by whole genome and transcriptomic sequencing data showing BK viral sequences in 21% of kidney transplant patients with bladder cancer [18]. In our study, the incidence of renal cell carcinoma was the highest followed by prostate cancer and urinary bladder carcinoma, although these proportions were not different between the groups.

We acknowledge certain limitations in our study. First, the standard of care screening protocol in practice today calls for more frequent monitoring of BK virus levels compared to the frequency of monitoring at the time of this study. Regardless, given the multiple checks within the first year of transplant, we believe that the screening intervals were short enough to capture BK viremia before it increased to levels high enough to cause BKVAN. This is further supported by the minority of BK viremic patients that developed BKVAN in our cohort. Second, our center has historically only performed indication biopsies for unexplained rise in serum creatinine or proteinuria. There is a possibility that subclinical rejection could have been missed in our study; nevertheless, graft and patient outcomes were not impacted. Third, the majority of the patients at our center received rATG as induction immunosuppression (84.2%). The findings in this single‐center study may not be generalizable to other centers that may use another form of induction agent such as alemtuzumab or basiliximab or no induction agent.

In summary, despite some earlier reports of worse kidney allograft and patient outcomes with BK viremia in the short‐ and intermediate‐term, we found that long‐term outcomes over a decade of follow‐up were similar when utilizing protocolized immunosuppression reduction strategies. Based on our study, a less frequent BK monitoring frequency may be non‐inferior to currently recommended guideline of more frequent BK monitoring. Since higher viral loads above 10 000 copies/mL of BK viremia are associated with poorer outcomes, early surveillance and detection with subsequent immunosuppression reduction to avoid BK virus levels from rising above this threshold remains key to avoiding adverse outcomes. Future prospectively designed multi‐center studies are needed to better define optimal BK virus screening intervals and the appropriate degree of immunosuppression reduction relative to BK viral loads without impacting outcomes.

## Conflicts of Interest

Jennifer Trofe's institution has received research funding from Veloxis Pharmaceuticals for a separate study initiative, none of which was used for the study design, performance, data analysis, or manuscript preparation of this study. Vishnu Potluri is supported by a career development grant from the National Institute of Diabetes and Digestive and Kidney Disease (K08DK127250). Vishnu Potluri received research grants to his institution from the Gift of Life Transplant Foundation and the Leonard Davis Institute of Health Economics to study organ donation and access to transplantation, respectively, which are unrelated to the work presented in this manuscript. Rest of the authors have no disclosures.

## Supporting information




**Supporting Figure 1:** Kaplan–Meier curves showing A) kidney graft survival, B) patient survival, and C) death‐censored graft survival among patients with different degrees of BK viremia versus BK never positive.
**Supporting Figure 2:** Scatterplot of BK viral load by quartiles.
**Supporting Figure 3:** Proportion of patients with DSA detection among BK ever positive and never positive groups.
**Supporting Figure 4:** Scatterplot showing the distribution of Class I and Class II DSA based on their MFI values over time.
**Supporting Table 1:** Standard maintenance immunosuppression protocol.
**Supporting Table 2:** Baseline characteristics of patients with BK viral loads above or below 10000 copies/ml and those who were never positive for BK virus.
**Supporting Table 3:** Multivariable Cox regression for death‐censored graft loss.
**Supporting Table 4:** Types of Class I DSA in descending order of frequency of detection.
**Supporting Table 5:** Type of Class II DSA in descending order of frequency of detection.
**Supporting Table 6:** Multivariable Cox regression for de novo DSA development.
**Supporting Table 7:** Multivariable Cox regression for acute rejection.
**Supporting Table 8:** Development of acute rejection episodes and de novo DSA development in patients without BK viremia and those with different degrees of BK viral load.
**Supporting Table 9:** Types of acute rejection episodes and treatments provided in those patients who had both de novo DSA and biopsy‐proven acute rejection.
**Supporting Table 10:** Development of de novo DSA in patients with BK viremia who had their anti‐metabolites held versus ones where they were not held.

## Data Availability

The data that support the findings of this study are available on request from the corresponding author. The data are not publicly available due to privacy or HIPAA restrictions.
