# Supplementary material for: Long‐Term Outcomes of BK Viremia in Kidney Transplant Recipients
Source: Clin Transplant. 2025 Oct 7;39(10):e70333. doi: 10.1111/ctr.70333 (PMC12503080; doi:10.1111/ctr.70333)
Supplement: Supplementary file 1 — Supporting Figure 1: Kaplan–Meier curves showing A) kidney graft survival, B) patient survival, and C) death‐censored graft survival among patients with different degrees of BK viremia versus BK never positive. Supporting Figure 2: Scatterplot of BK viral load by quartiles. Supporting Figure 3: Proportion of patients with DSA detection among BK ever positive and never positive groups. Supporting Figure 4: Scatterplot showing the distribution of Class I and Class II DSA based on their MFI values over time. Supporting Table 1: Standard maintenance immunosuppression protocol. Supporting Table 2: Baseline characteristics of patients with BK viral loads above or below 10000 copies/ml and those who were never positive for BK virus. Supporting Table 3: Multivariable Cox regression for death‐censored graft loss. Supporting Table 4: Types of Class I DSA in descending order of frequency of detection. Supporting Table 5: Type of Class II DSA in descending order of frequency of detection. Supporting Table 6: Multivariable Cox regression for de novo DSA development. Supporting Table 7: Multivariable Cox regression for acute rejection. Supporting Table 8: Development of acute rejection episodes and de novo DSA development in patients without BK viremia and those with different degrees of BK viral load. Supporting Table 9: Types of acute rejection episodes and treatments provided in those patients who had both de novo DSA and biopsy‐proven acute rejection. Supporting Table 10: Development of de novo DSA in patients with BK viremia who had their anti‐metabolites held versus ones where they were not held. [file CTR-39-e70333-s001.docx]

**Supplementary Figure 1. Kaplan-Meier curves showing A) kidney graft survival, B) patient survival, and C) death-censored graft survival among patients with different degrees of BK viremia versus BK never positive**

**Supplementary Figure 2. Scatterplot of BK viral load by quartiles**

**Supplementary Figure 3. Proportion of patients with DSA detection among BK ever positive and never positive groups.**

**Supplementary Figure 4. Scatterplot showing the distribution of Class I and Class II DSA based on their MFI values over time.**

**Supplementary Table 1. Standard maintenance immunosuppression protocol**

**Supplementary Table 2. Baseline characteristics of patients with BK viral loads above or below 10000 copies/ml and those who were never positive for BK virus.**

**Supplementary Table 3. Multivariable Cox regression for death-censored graft loss.**

**Supplementary Table 4. Types of Class I DSA in descending order of frequency of detection**

**Supplementary Table 5. Type of Class II DSA in descending order of frequency of detection**

**Supplementary Table 6. Multivariable Cox regression for de novo DSA development**

**Supplementary Table 7. Multivariable Cox regression for acute rejection**

**Supplementary Table 8. Development of acute rejection episodes and de novo DSA development in patients without BK viremia and those with different degrees of BK viral load**

**Supplementary Table 9. Types of acute rejection episodes and treatments provided in those patients who had both de novo DSA and biopsy-proven acute rejection**

**Supplementary Table 10. Development of de novo DSA in patients with BK viremia who had their anti-metabolites held versus ones where they were not held**

**Supplementary Figure 1.** Kaplan-Meier curves showing A) kidney graft survival, B) patient survival, and C) death-censored graft survival among patients with different degrees of BK viremia versus BK never positive

A.


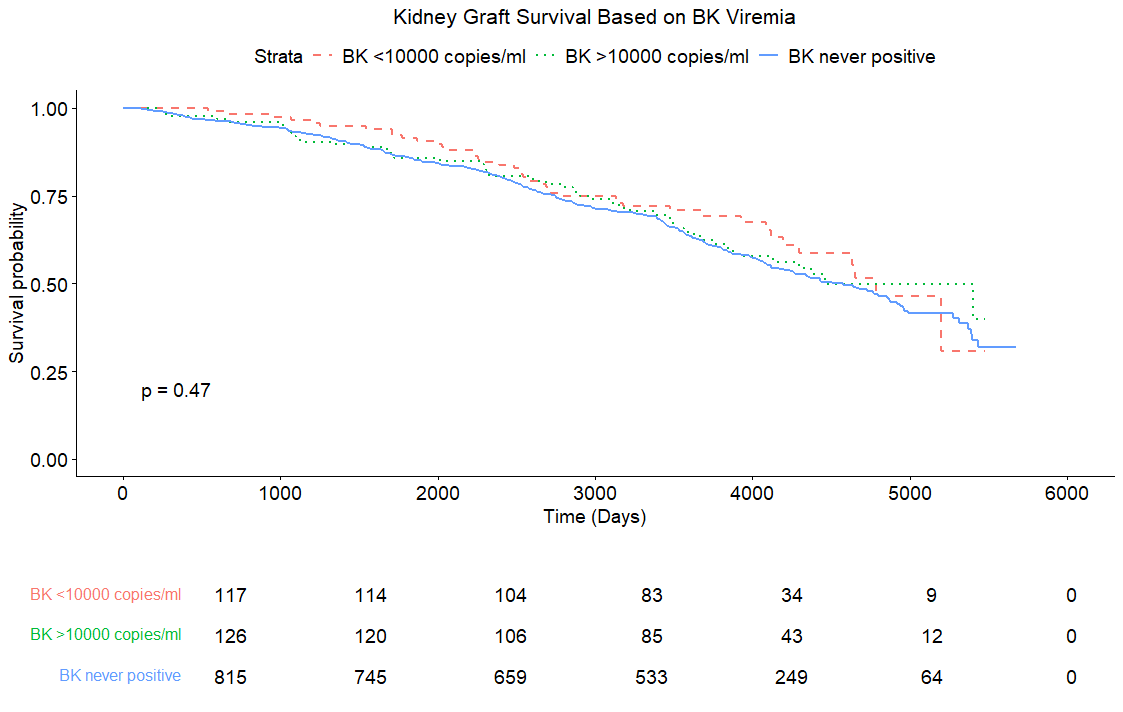


B.


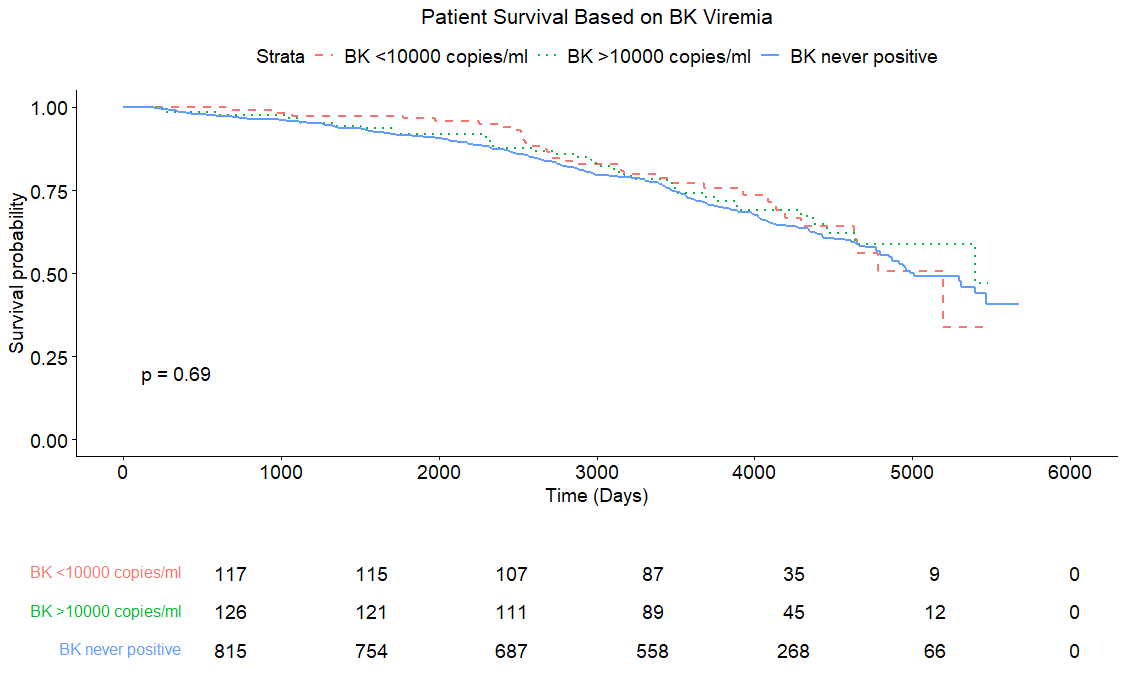


C.


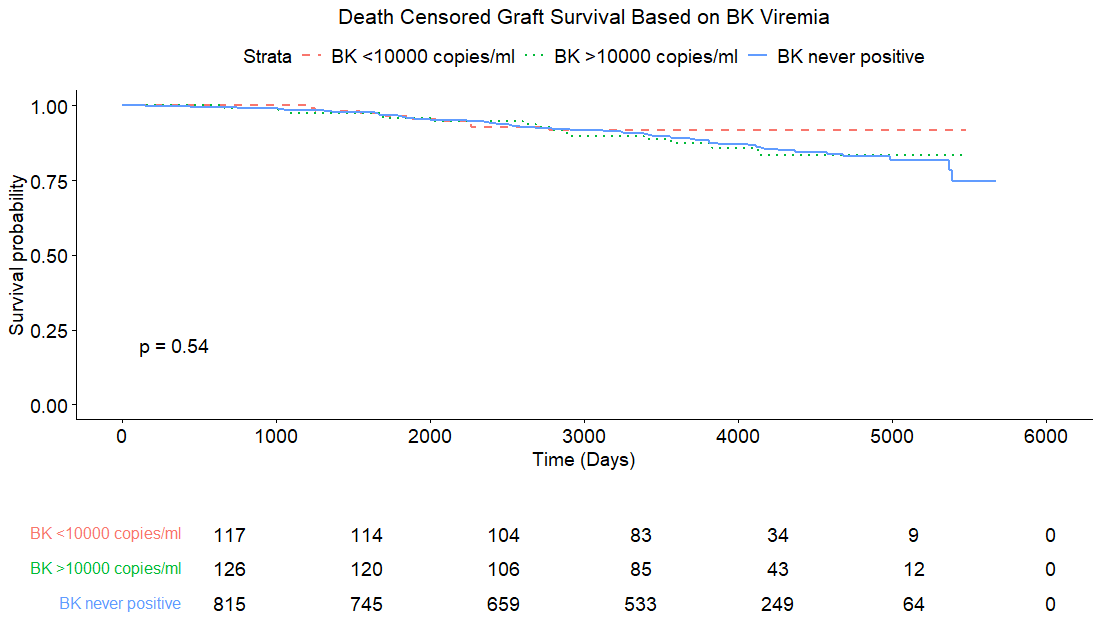


**Supplementary Figure 2.** Scatterplot of BK viral load by quartiles


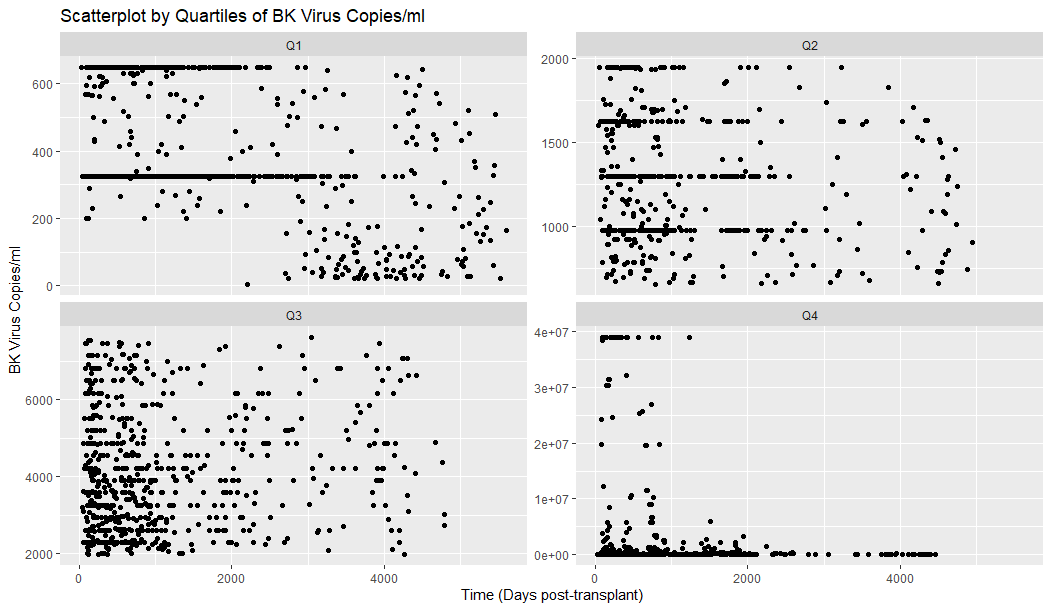


**Supplementary Figure 3.** Proportion of patients with DSA detection among BK ever positive and never positive groups.


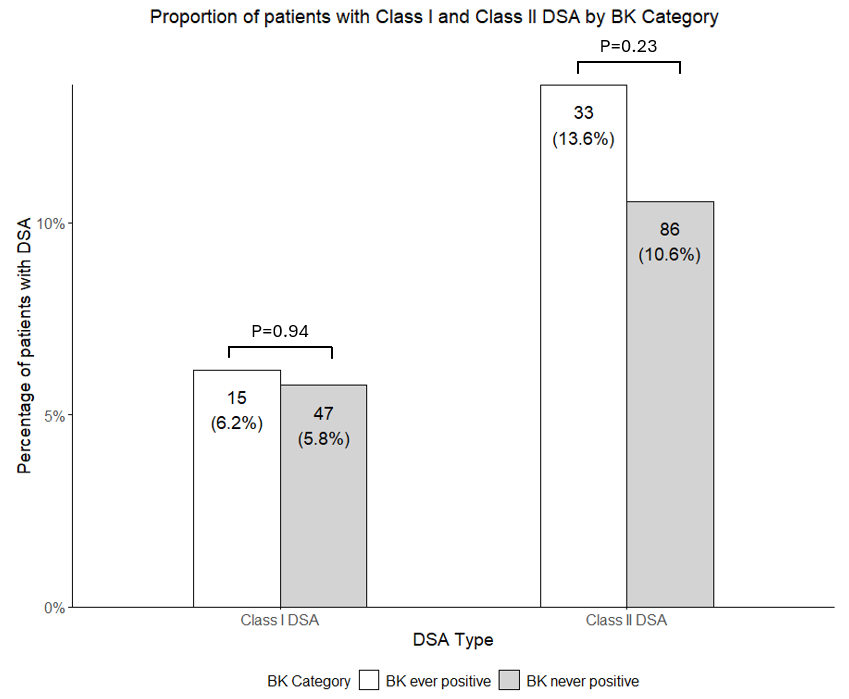


**Supplementary Figure 4.** Scatterplot showing the distribution of Class I and Class II DSA based on their MFI values over time.


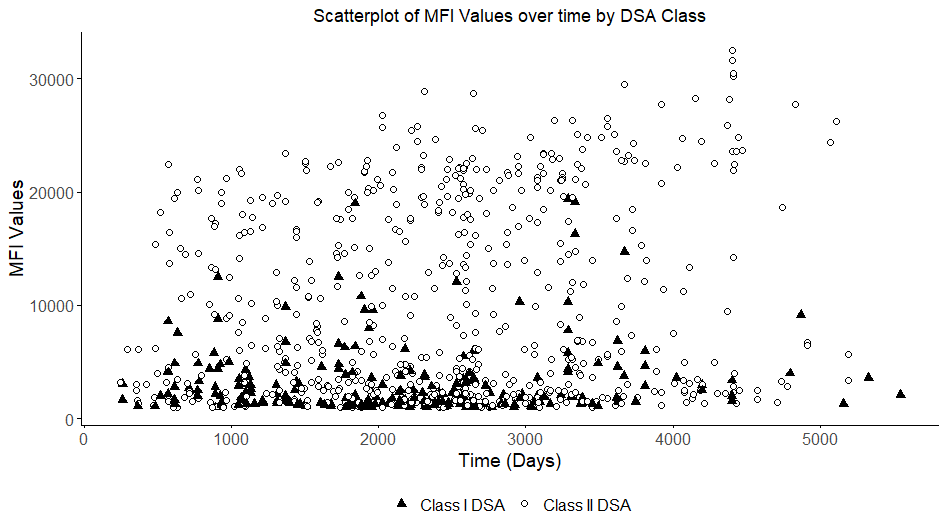


**Supplementary Table 1.** Standard maintenance immunosuppression protocol

| **Immunosuppressive agent** | **Time post-transplant** | **Target level** |
| --- | --- | --- |
| Tacrolimus | Week 0-12 | 8-12 ng/ml |
|  | Week 12-24 | 6-8 ng/ml |
|  | Week 24-48 | 5-7 ng/ml |
|  | Beyond Week 48 | 4-6 ng/ml |
|  |  |  |
| Cyclosporine | Week 0-12 | 150-250 ng/ml |
|  | Week 12-24 | 150-200 ng/ml |
|  | Week 24-48 | 100-200 ng/ml |
|  | Beyond Week 48 | 50-100 ng/ml |
|  |  |  |
| Mycophenolate mofetil | Standard dosing from POD 0 | 500 mg twice a day |
|  |  |  |
| Mycophenolate sodium | Standard dosing from POD 0 | 360 mg twice a day |
|  |  |  |
| Prednisone tapering protocol | Post op day 0 | Methylprednisolone 500 mg |
|  | Post op day 1 | Methylprednisolone 250 mg |
|  | Post op day 2 | Methylprednisolone 125 mg |
|  | Post op day 3 | Methylprednisolone 100 mg |
|  | Post op day 4 | Prednisone 80 mg |
|  | Post op day 5 | Prednisone 60 mg |
|  | Post op day 6 | Prednisone 40 mg |
|  | Post op day 7 | Prednisone 20 mg |
|  | Post op day 8-9 | Prednisone 10 mg |
|  | Post op day 10 onwards | Prednisone 5 mg |

**Supplementary Table 2.** Baseline characteristics of patients with BK viral loads above or below 10000 copies/ml and those who were never positive for BK virus

| **Characteristics ^¶^** | **BK Never Positive (N=815)** | **BK viral load <10000 copies/ml (N=117)** | **BK viral load >10000 copies/ml (N=126)** | **p value** |
| --- | --- | --- | --- | --- |
| **Recipient characteristics** |  |  |  |  |
| Age | 50.6 (13.3) | 53 (12.3) | 52.1 (12.1) | 0.11 |
| Race |  |  |  | 0.15 |
| - African American | 541 (66.4%) | 67 (57.3%) | 82 (65.1%) |  |
| - Non-African American | 274 (33.6%) | 50 (42.7%) | 44 (34.9%) |  |
| Female Gender | 320 (39.3%) | 47 (40.2%) | 46 (36.5%) | 0.81 |
| Diabetes Mellitus | 282 (34.6%) | 43 (36.8%) | 37 (29.4%) | 0.61 |
| BMI | 27.1 (5.2) | 27.8 (5.7) | 27.4 (4.9) | 0.28 |
| Recipient Blood Type |  |  |  | 0.15 |
| - A | 321 (39.4%) | 40 (34.2%) | 40 (31.7%) |  |
| - AB | 43 (5.3%) | 6 (5.1%) | 6 (4.8%) |  |
| - B | 126 (15.5%) | 13 (11.1%) | 15 (11.9%) |  |
| - O | 325 (39.9%) | 58 (49.6%) | 65 (51.6%) |  |
| Rabbit anti-thymocyte globulin induction | 674 (82.7%) | 105 (89.7%) | 112 (88.9%) | 0.05 |
| Peak PRA Class 1 | 46.2 (34.4) | 35.485 (36.8) | 49.9 (36.6) | 0.20 |
| Peak PRA Class 2 | 56.3 (31.5) | 60.300 (33.7) | 75.2 (25.3) | 0.06 |
| **Donor characteristics** |  |  |  |  |
| Age | 38.8 (15.7) | 36.4 (15.7) | 38.7 (15.3) | 0.29 |
| Race |  |  |  | < 0.001 |
| - African American | 103 (12.6%) | 30 (25.6%) | 22 (17.5%) |  |
| - Non-African American | 712 (87.4%) | 87 (74.4%) | 104 (82.5%) |  |
| Female  Gender | 397 (48.7%) | 48 (41.0%) | 60 (47.6%) | 0.29 |
| Diabetes Mellitus | 58 (7.1%) | 9 (7.7%) | 13 (10.3%) | 0.41 |
| BMI | 26.7 (6.5) | 26.9 (6.8) | 26.7 (5.6) | 0.95 |
| Hypertension | 148 (18.2%) | 21 (17.9%) | 26 (20.6%) | 0.59 |
| Terminal Creatinine | 1.08 (0.61) | 1.09 (0.55) | 1.12 (0.63) | 0.85 |
| HCV Antibody Positive | 0 (0.0%) | 1 (0.9%) | 0 (0.0%) | 0.19 |
| Dialysis at Time of Transplant |  |  |  | 0.51 |
| Not on Dialysis | 172 (21.1%) | 25 (21.4%) | 21 (16.7%) |  |
| On Dialysis | 643 (78.9%) | 92 (78.6%) | 105 (83.3%) |  |
| Cause of death in donor |  |  |  | 0.76 |
| -  Anoxia | 194 (34.7%) | 35 (40.2%) | 25 (27.2%) |  |
| -  Cerebrovascular/stroke | 171 (30.6%) | 25 (28.7%) | 33 (35.9%) |  |
| -  Head trauma | 184 (32.9%) | 26 (29.9%) | 32 (34.8%) |  |
| -  CNS tumor | 3 (0.5%) | 0 (0.0%) | 0 (0.0%) |  |
| -  Others | 7 (1.3%) | 1 (1.1%) | 2 (2.2%) |  |
| Donor Blood Type |  |  |  | 0.8 |
| -  A | 288 (35.3%) | 33 (28.2%) | 34 (27%) |  |
| -  AB | 22 (2.7%) | 4 (3.5%) | 3 (2.4%) |  |
| -  B | 100 (12.3%) | 11 (9.4%) | 14 (11.1%) |  |
| -  O | 405 (49.7%) | 69 (59.0%) | 75 (59.5%) |  |
| Kidney Donor Profile Index |  |  |  | 0.85 |
| - 0-20% | 148 (26.6%) | 26 (29.9%) | 28 (30.4%) |  |
| - 21-35% | 94 (16.9%) | 18 (20.7%) | 15 (16.3%) |  |
| - 35-85% | 279 (50.2%) | 40 (46.0%) | 44 (47.8%) |  |
| - 86-100% | 35 (6.3%) | 3 (3.4%) | 5 (5.4%) |  |
| **Transplant characteristics** |  |  |  |  |
| Cold Ischemia Time (h) | 13.2 (6.8) | 12.4 (6.4) | 13.5 (7) | 0.45 |
| HLA Mismatch |  |  |  | 0.09 |
| -  0 | 85 (10.4%) | 7 (6.0%) | 6 (4.8%) |  |
| -  1 | 15 (1.8%) | 1 (0.9%) | 0 (0.0%) |  |
| -  2 | 56 (6.9%) | 9 (7.7%) | 5 (4.0%) |  |
| -  3 | 147 (18.0%) | 19 (16.2%) | 16 (12.7%) |  |
| -  4 | 181 (22.2%) | 31 (26.5%) | 41 (32.5%) |  |
| -  5 | 224 (27.5%) | 37 (31.6%) | 41 (32.5%) |  |
| -  6 | 107 (13.1%) | 13 (11.1%) | 17 (13.5%) |  |
|  |  |  |  |  |

**Supplementary Table 3.** Multivariable Cox regression for death-censored graft loss.


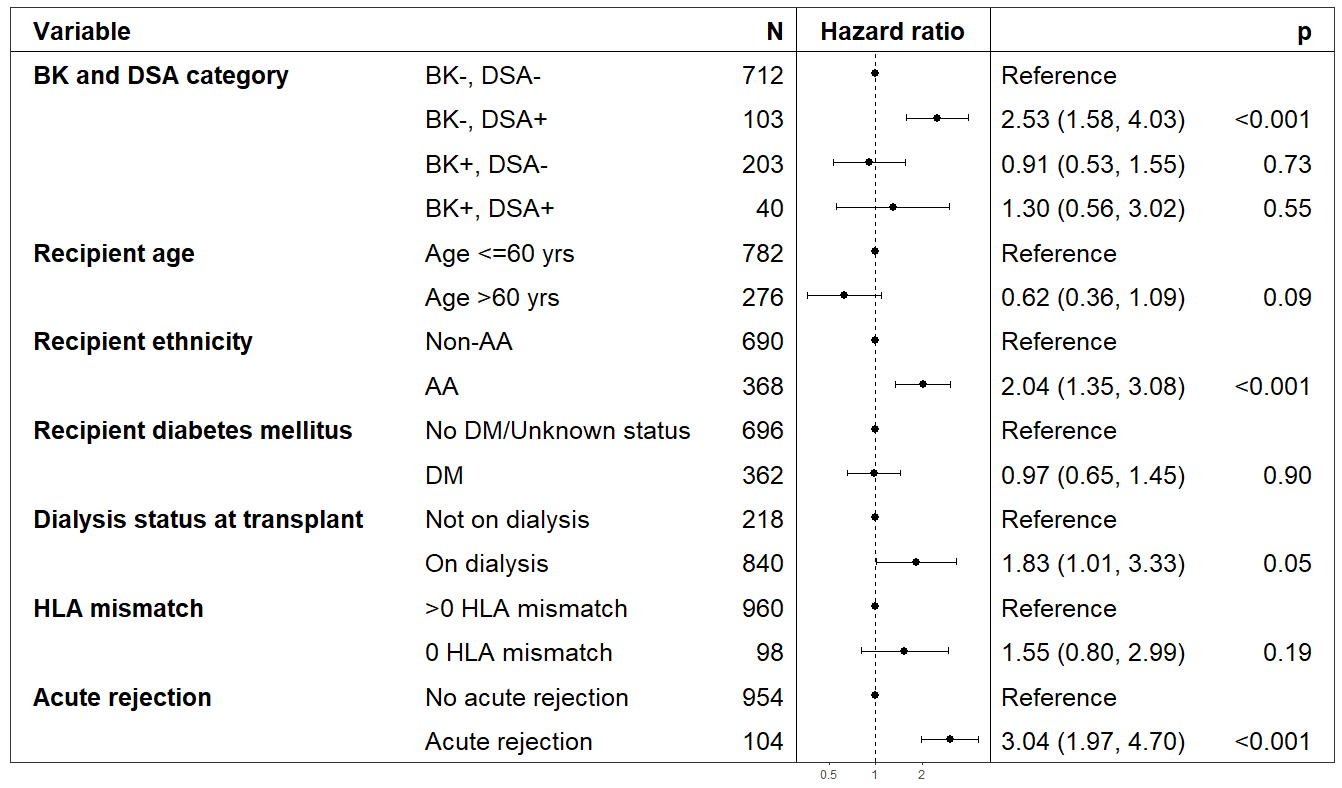


**Supplementary Table 4.** Types of Class I DSA in descending order of frequency of detection

| **Class I DSA** | **Frequency of detection** |
| --- | --- |
| A2 | 94 |
| B44 | 92 |
| A1 | 79 |
| B8 | 71 |
| DSABwUNKNOWN | 51 |
| Cw4 | 49 |
| B51 | 44 |
| B57 | 34 |
| B7 | 34 |
| Cw5 | 32 |
| A24 | 29 |
| B35 | 23 |
| A11 | 20 |
| A3 | 20 |
| Cw2 | 20 |
| Cw7 | 20 |
| Cw10 | 19 |
| Cw6 | 19 |
| A30 | 16 |
| A31 | 16 |
| B27 | 15 |
| B49 | 14 |
| B63 | 12 |
| A23 | 11 |
| A68 | 11 |
| B62 | 11 |
| Cw1 | 11 |
| A25 | 10 |
| B45 | 10 |
| B75 | 10 |
| C4 | 10 |
| Cw8 | 10 |
| B41 | 9 |
| B53 | 9 |
| A26 | 8 |
| B13 | 8 |
| B58 | 8 |
| B60 | 8 |
| B65 | 8 |
| C*16 | 8 |
| Cw9 | 8 |
| B18 | 7 |
| B38 | 7 |
| B52 | 7 |
| C9 | 7 |
| B55 | 6 |
| C*14 | 6 |
| C5 | 6 |
| C7 | 6 |
| A29 | 5 |
| A33 | 5 |
| B71 | 5 |
| C2 | 5 |
| A32 | 4 |
| B*44:03 | 4 |
| B39 | 4 |
| C12 | 4 |
| C14 | 4 |
| A*24:02 | 3 |
| A74 | 3 |
| B48 | 3 |
| B56 | 3 |
| B61 | 3 |
| B78 | 3 |
| Cw14 | 3 |
| A*66:01 | 2 |
| A36 | 2 |
| B*51:01 | 2 |
| B47 | 2 |
| C*12 | 2 |
| C*17 | 2 |
| C16 | 2 |
| C6 | 2 |
| Cw16 | 2 |
| Cw17 | 2 |
| B37 | 1 |
| B42 | 1 |
| B50 | 1 |
| B51:01 | 1 |
| Bw4 | 1 |
| C17 | 1 |
| C18 | 1 |
| Cw15 | 1 |
| C10 | 1 |

**Supplementary Table 5.** Type of Class II DSA in descending order of frequency of detection

| **Class II DSA** | **Frequency of detection** |
| --- | --- |
| DQ7 | 416 |
| DR53 | 246 |
| DQ8 | 206 |
| DQ2 | 204 |
| DQ5 | 186 |
| DQA1*05 | 177 |
| DQ6 | 130 |
| DQA1*03 | 99 |
| DQ4 | 83 |
| DQ9 | 81 |
| DR51 | 74 |
| DR52 | 45 |
| DR4 | 35 |
| DR7 | 34 |
| DPB1*03:01 | 33 |
| DQA1*03:02 | 32 |
| DR12 | 25 |
| DPB1*04:01 | 22 |
| DQA1*04 | 22 |
| DR15 | 21 |
| DQA05 | 19 |
| DQA1*02:01 | 18 |
| DQ3 | 17 |
| DPB1*02:01 | 16 |
| DPB1*04:02 | 15 |
| DQB1*06:03 | 14 |
| DR9 | 14 |
| DR13 | 13 |
| DQA*05 | 11 |
| DR11 | 11 |
| DR14 | 11 |
| DPA1*01:03 | 9 |
| DPA1*02 | 9 |
| DPB1*17:01 | 9 |
| DQA1*03:03 | 9 |
| DQB1*05:01 | 9 |
| DQA 05 | 8 |
| DR1 | 8 |
| DR10 | 7 |
| DRB3*02:02 | 7 |
| DRB5*01:01 | 7 |
| DP2 | 6 |
| DPA1*01 | 6 |
| DQA1*02 | 6 |
| DR8 | 5 |
| DRB3*01 | 5 |
| DPA 02 | 4 |
| DQA 02 | 4 |
| DQA*03 | 4 |
| DQA1*05:01 | 4 |
| DR103 | 4 |
| DP 10:01 | 3 |
| DP11 | 3 |
| DPB1*105:01 | 3 |
| DQA 05:01 | 3 |
| DQA03 | 3 |
| DQA1*04:01 | 3 |
| DQA1*05/06 | 3 |
| DR16 | 3 |
| DRB5*01 | 3 |
| DP4 | 3 |
| DP3 | 2 |
| DPA1*04 | 2 |
| DPB 04:01 | 2 |
| DPB1* 04:02 | 2 |
| DPB1*01:01 | 2 |
| DPB1*02 | 2 |
| DPB1*11 | 2 |
| DQ7-DQA1*05 | 2 |
| DQ8-DQA1*03 | 2 |
| DQA 03 | 2 |
| DQA:0201 | 2 |
| DQA1*05:05 | 2 |
| DQB1*06:04 | 2 |
| DRB3*01:01 | 2 |
| DRB3*02 | 2 |
| DP 0401 | 1 |
| DP1 | 1 |
| DPA 01 | 1 |
| DPA01 | 1 |
| DPA1*03 | 1 |
| DPB 02:01 | 1 |
| DPB 0402 | 1 |
| DPB 104:01 | 1 |
| DPB02:01 | 1 |
| DPB1* 04 | 1 |
| DPB1*03:01 | 1 |
| DPB1*09 | 1 |
| DPB1*11:01 | 1 |
| DPB1*131:01 | 1 |
| DPB1*17 | 1 |
| DPB1*18:01 | 1 |
| DQ 8 | 1 |
| DQ05 | 1 |
| DQ6 | 1 |
| DQ7 | 1 |
| DQA 02:01 | 1 |
| DQA04 | 1 |
| DQA1*03:01 | 1 |
| DQA1*03:02/03 | 1 |
| DQA5 | 1 |
| DQB1*05 | 1 |
| DQB1*06:02 | 1 |
| DQB1*06:09 | 1 |
| DR17 | 1 |
| DRB1*14:54 | 1 |
| DRw53 | 1 |
| DP 0402 | 1 |
| DPB04:02 | 1 |
| DPB1*14:01 | 1 |
| DQ 7 | 1 |

**Supplementary Table 6.** Multivariable Cox regression for *de novo* DSA development


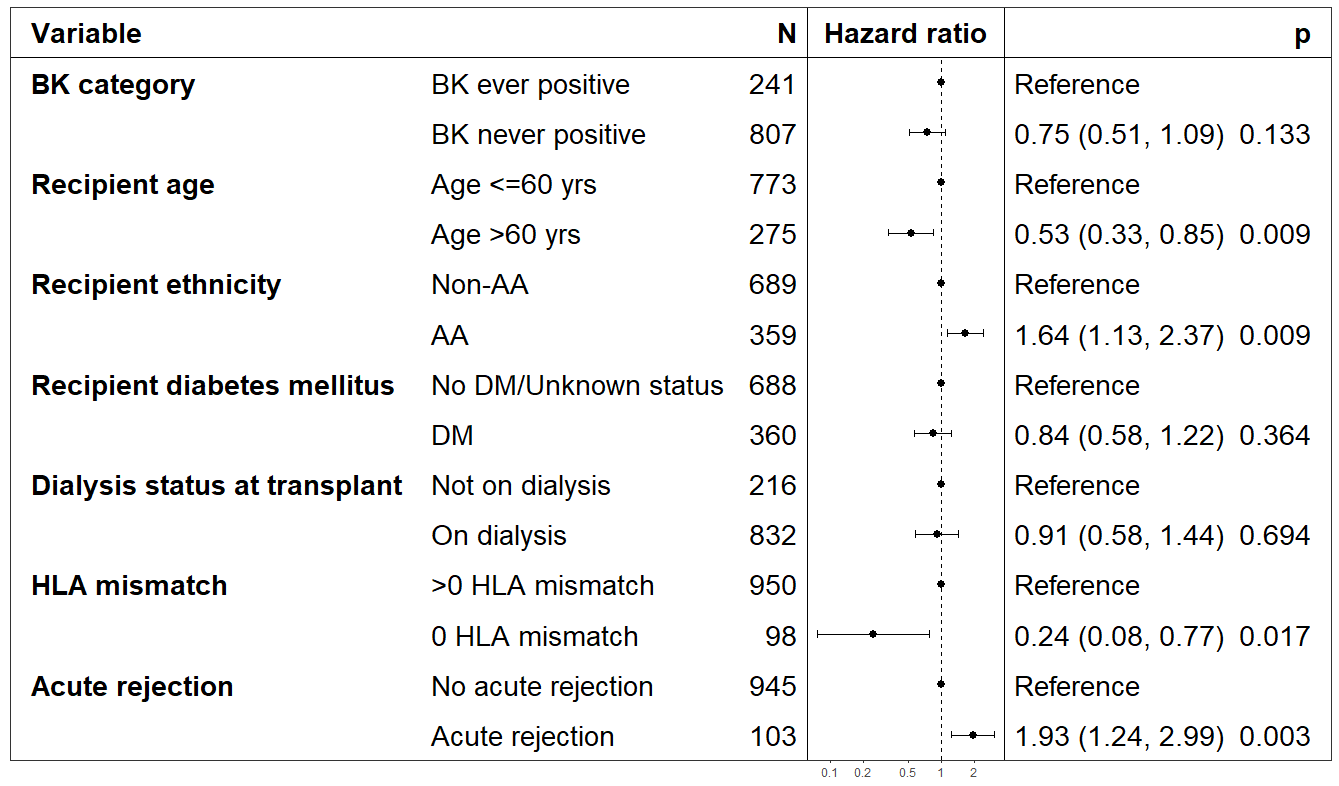


**Supplementary Table 7.** Multivariable Cox regression for acute rejection


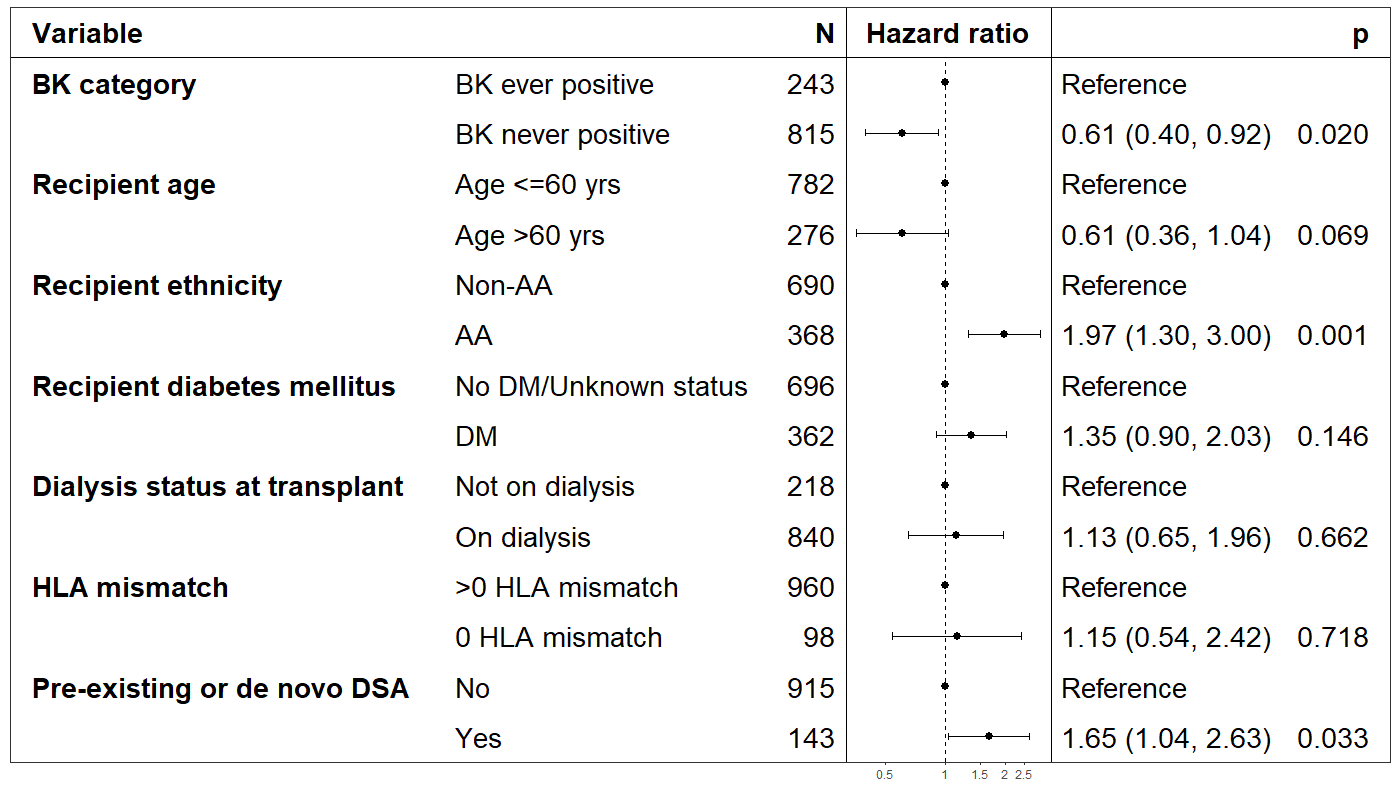


**Supplementary Table 8.** Development of acute rejection episodes and de novo DSA development in patients without BK viremia and those with different degrees of BK viral load

|  | Acute rejection episodes^§^ | | P value | De novo DSA development^†^ | | P value |
| --- | --- | --- | --- | --- | --- | --- |
|  | No | Yes | 0.02 | No | Yes | 0.07 |
| BK never positive | 744 (91.3%) | 71 (8.7%) |  | 712 (88.2%) | 95 (11.8%) |  |
| BK <10,000 copies/ml | 105 (89.7%) | 12 (10.3%) |  | 101 (87.8%) | 14 (12.2%) |  |
| BK >10,000 copies/ml | 105 (83.3%) | 21 (16.7%) |  | 102 (80.9%) | 24 (19.1%) |  |

^§^ Includes all 1058 patients

^†^ Includes only 1048 with de novo DSAs excluding 10 patients with pre-existing DSAs.

**Supplementary Table 9.** Types of acute rejection episodes and treatments provided in those patients who had both de novo DSA and biopsy-proven acute rejection

| **Acute rejection type** | **Number of patients** | **Treatment** |
| --- | --- | --- |
| **T cell mediated rejection** |  |  |
| -Borderline rejection | 6 | Methylprednisolone in 3 patients  No treatment in 3 patients |
| -Banff Grade 1a rejection | 1 | rATG x 3 doses |
| -Banff Grade 1b rejection | 6 | Methylprednisolone and rATG in 6 patients |
| -Banff Grade 2 rejection | 2 | rATG and methylprednisolone in 2 patients |
| -Banff Grade not specified | 1 | No treatment |
|  |  |  |
| **Antibody-mediated rejection** | 2 | Methylprednisolone, rATG, IVIG and plasmapheresis in 1 patient  No treatment in 1 patient |
|  |  |  |
| **Mixed T cell and antibody mediated rejection** |  |  |
| -Borderline rejection | 2 | Methylprednisolone, rATG, IVIG, plasmapheresis in 2 patients |
| -Banff Grade 1a rejection | 1 | Methylprednisolone in 1 patient |
| -Banff Grade 1b rejection | 3 | Methylprednisolone, rATG, IVIG, plasmapheresis in 2 patients  Methylprednisolone, rATG, plasmapheresis in 1 patient |
| -Banff Grade 3 rejection | 1 | Methylprednisolone, IVIG, plasmapheresis in 2 patients |
| -Banff Grade not specified | 1 | Methylprednisolone, rATG, IVIG, plasmapheresis in 1 patient |

**Supplementary Table 10.** Development of de novo DSA in patients with BK viremia who had their anti-metabolites held versus ones where they were not held.

|  | De novo DSA | No de novo DSA | P value |
| --- | --- | --- | --- |
| Antimetabolite held | 26 (19%) | 111 (81%) | 0.3 |
| Antimetabolite not held | 14 (13.2%) | 92 (86.8%) |  |
